# Computational Analysis of Thermal Performance of Heat Sinks with Foam Structures

**DOI:** 10.3390/ma18235280

**Published:** 2025-11-22

**Authors:** Welteji Iticha, Tomasz Stręk

**Affiliations:** Institute of Applied Mechanics, Poznan University of Technology, Jana Pawla II 24, 60-965 Poznan, Poland

**Keywords:** heat sink, TPMS, porous structure, fluid flow, heat transfer

## Abstract

Ensuring efficient heat transfer to maintain optimal system performance is crucial in modern electronics owing to the rise of artificial intelligence. In the last few decades, scholars have explored various strategies for enhancing electronic device thermal management, focusing on the effects of fin shape, dimension, and spacing on heat transfer efficiency. Recent advancements in additive manufacturing have enabled fabrication of complex geometries, such as triply periodic minimal surfaces (TPMSs), which represent promising alternatives to conventional designs. This study presents a comparative analysis of the thermal performance and fluid flow characteristics of two foam TPMS-based (gyroid and primitive) heat sinks with wavy fins made using aluminum foam. COMSOL Multiphysics version 5.1, employed along with the implemented finite element method, was used to simulate convective heat transfer, pressure drop, the Nusselt number, and thermal performance at different fluid velocities along the length of a channel. The foam structure was heated by a copper plate, and the Nusselt number was evaluated over porosity levels from 0.1 to 0.9. A porosity between 0.5 and 0.7 offers the best balance of cooling performance and pumping power. Foam TPMS heat sinks, particularly those with a gyroid structure, provide enhanced thermal dissipation owing to their high surface area-to-volume ratio and interconnected geometry. Our findings confirm that TPMS heat sinks have promising potential for use as alternatives to conventional wavy designs for advanced thermal management applications.

## 1. Introduction

Advancements in electronics, aerospace, the automotive industry, and other industrial sectors necessitate compact and more efficient heat dissipation devices. A heat sink (HS) device dissipates heat from the source into the environment either passively (naturally) or actively (using rotating elements such as fans and pumps) to regulate temperature and prevent overheating. The performance of electronic devices is directly limited by their ability to dissipate heat, and inadequate heat reduction ultimately degrades performance, driving the development of more advanced HSs [1]. Several factors, including vibration, humidity, temperature, and dust, contribute to electronic equipment failure. Thermal fatigue accounts for over 40% of cases of device failure, making effective heat removal essential for long-term durability [2]. Researchers have investigated various mechanisms for enhancing heat transfer in heat sinks. In a conventional HS model, fin geometry, arrangement, spacing, and size greatly determine thermal performance [3,4,5]. The thermal performance of heat sinks with rectangular, elliptical, cylindrical, and plate fin geometries depends on flow conditions and design constraints. Staggered fin arrangements, particularly for elliptical designs, outperform inline arrangements in terms of heat transfer efficiency [6]. Perforated fins with cavities further improve performance relative to regular fins, as the holes promote heat dissipation [2]. Fins’ spaces and thickness also significantly influence overall performance. Reduced fin spacing enhances efficiency in water-cooled mini-channel heat sinks, while thinner fins lower temperature rise [7,8]. Nevertheless, conventional heat sink designs still fail to meet the desired performance requirements regarding compactness and high thermal efficiency in advanced thermal systems.

**Figure 1:**
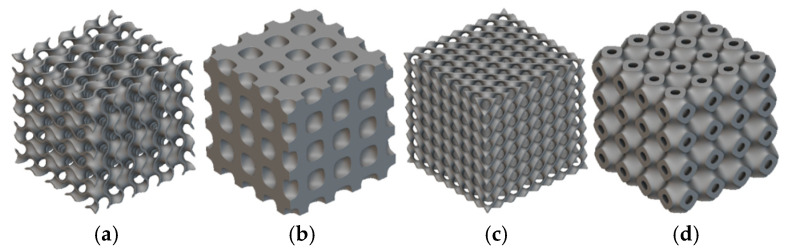
Types of TPMS lattice structures: (**a**) gyroid, (**b**) neovius, (**c**) diamond, and (**d**) Schwarz [19].

For many years, cellular, lattice, foam, and architectural materials and structures have been used in engineering [9], biomedicine [10], and other industries due to their lightness, high-strength-to-weight ratio [11], and tunable mechanical and thermal properties. They have a wide range of applications, including energy absorption [12], thermal insulation or management [13,14], cores in sandwich panels, and implants or prostheses [10]. Triply periodic minimal surface (TPMS) structures have recently drawn a great deal of attention with respect to HS applications due to their significant thermophysical properties and compact design. These structures have a high surface area-to-volume ratio, which is the basic requirement for thermal management. Such inherent qualities make TPMS structures highly effective in addressing challenges related to heat dissipation [8]. Triply periodic minimal surfaces (TPMSs) are non-self-intersecting periodic implicit surfaces with a mean curvature of zero, meaning that the curvature along the principal plane is equal and opposite at every point, making the mean curvature zero. These surfaces are commonly found in natural structures, such as butterfly wings and the weevil Lamprocyphus augustus [15,16]. TPMS lattices are classified into sheets and networks based on their internal pore connectivity. A network lattice is a continuous skeleton throughout its entire volume, and a sheet lattice is a continuous thin sheet that separates two distinct and interconnected porous regions [17,18]. Figure 1 illustrates different TPMS lattice configurations. The intricate internal channel of a TPMS allows for a greater heat transfer area, which enhances heat transfer.

Several scholars have investigated the effects of different TPMS design parameters on the overall heat transfer performance of heat sinks. Liu et al. (2025) [20] compared and reported the fluid flow and heat transfer characteristics of a traditional rectangular fin (RF) with a TPMS lattice fabricated from AlSi10Mg alloys. The results revealed that the TPMS lattice provided a larger convective heat transfer area and had a more complex internal configuration, which enhanced the frictional resistance of the fluid and increased convective heat transfer coefficient under the same flow conditions. A numerical method was employed to evaluate the heat transfer and fluid flow characteristics of an HS based on TPMS structures, including diamond-solid, gyroid-solid, and gyroid-sheet configurations, under natural free-convection conditions [21]. Among the tested geometries, the gyroid-sheet configuration exhibited superior thermal efficiency when both the top and bottom surfaces were open. TPMS-based designs led to a 35–50% improvement in performance relative to traditional pin–fin heat sinks. Attarzadeh et al. (2021) [15] analyzed the contribution of TPMS thickness to determine the thermal efficiency of diamond-sheet structures. The results indicated that increasing wall thickness initially boosted the convective heat transfer coefficient. However, exceedingly thick walls negatively impacted fluid flow, ultimately diminishing overall thermal performance. Lattice cell size is another parameter determining fluid flow characteristics. A decrease in cell size from 10 mm to 6 mm increased the pressure drop across the diagonal cross-section by 63.1%, rising from 1.004 kPa to 1.632 kPa [22]. Beer and Rybár [23] conducted a comparative analysis of sheet, skeletal, and combined gyroid structures based on numerical simulations of fluid flow and heat transfer. The combined gyroid structure demonstrated the highest heat transfer efficiency, reflected in superior Nusselt numbers and Chilton–Colburn j-factors. A comparison of the convective heat transfer enhancement of gyroid and diamond TPMS structures was conducted using the deform control parameter (β), which varied from 0.1 to 0.75, based on numerical and experimental analysis. The results revealed that increasing β values also increased the convective heat transfer coefficient in the gyroid and diamond structures, while a greater increment was observed in the gyroid structure [24]. The convective properties of a TPMS are sometimes accompanied by recursive or fractal structural characteristics [25]. The results of one study revealed a significant reduction in flow resistance for a TPMS with first-order fractal characteristics relative to the original TPMS structure. However, second-order fractal characteristics led to a substantial increase in flow resistance. Tang et al. [26] developed a TPMS with fin structures (TFS-gyroid), showing that adding fins to a gyroid TPMS enhances convective heat transfer by 27–34%, while the pressure drop from inlet to outlet increased up to six-fold depending on fin height. Subsequently, Al-Ketan et al. [27] discovered that increasing wall thickness in the flow direction in TPMS structures simultaneously decreases both the convective heat transfer coefficient and channel resistance. 

In this paper, we aim to evaluate the fluid flow and heat transfer characteristics of two foam TPMS structures (gyroid and primitive) and a traditional wavy fin in a heat sink using numerical methods. All structures were constructed using aluminum foam material with a porosity range of 0.1 to 0.9. Finite element analysis was used to solve governing equations with boundary conditions. The foam structure was heated by a copper plate, and the Nusselt number was evaluated across different porosity levels. We found that a porosity of between 0.5 and 0.7 offers the best balance of cooling performance and pumping power. The heat sinks with a foam TPMS structure, particularly a gyroid structure, allowed increased thermal dissipation owing to their high surface area-to-volume ratio and interconnected geometry. The results confirm the potential of foam TPMS heat sinks for use as alternatives to conventional wavy designs for advanced thermal management applications.

Heat sinks are typically made of aluminum or copper and have fins, pins, or other elements that increase the surface area of the component to improve and accelerate the transfer of heat to the surrounding environment (e.g., fluid or air). Usually, these elements are made of solid material, most often metal with high thermal conductivity. The main novelty of this study is our analysis of the thermal performance of heat sinks with foam TPMS and foam conventional wavy structures. For comparison, two TPMS structures (gyroid and primitive) and wavy-fin conventional heat sink geometries were selected. All structures (TPMS and wavy) were composed of aluminum foam material with a porosity ranging from 0.1 to 0.9. Finite element analysis was used to solve governing equations with boundary conditions. It is shown that the porosity of the structure influences the thermal properties of the heat sink.

This paper consists of four sections. The first presents an introduction and the state of the art. The second is devoted to materials and methods, mathematical equations of TPMS lattices geometries, geometric modeling, governing equations with boundary conditions, and the influence of mesh elements on the computational results. The third section explains the numerical results and provides a discussion. The last section presents the conclusions derived from the computational analysis.

## 2. Materials and Methods

### 2.1. Mathematical Equations of TPMS Lattices

**Figure 2:**
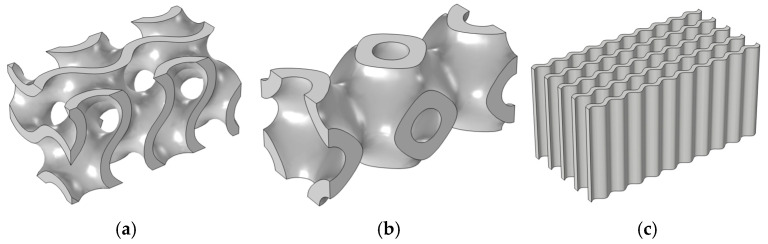

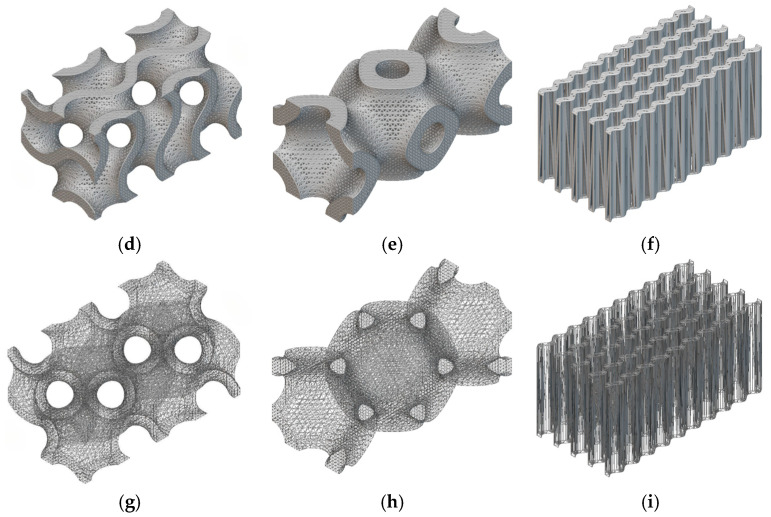
Three-dimensional models of heat sink structures: computational solid domains of (**a**) gyroid, (**b**) primitive, and (**c**) wavy structures and examples of the porous domain of (**d**,**g**) gyroid, (**e**,**h**) primitive, and (**f**,**i**) wavy structures with different porosities.

A triply periodic minimal surface (TPMS) is a minimal surface that extends periodically in three spatial directions. It is designed using mathematical algorithms based on an implicit function. The level-set equations are mathematical equations that describe a three-dimensional surface. This surface is defined by a trigonometric function, ϕ(x,y,z), where all points (x,y,z) on the surface satisfy the condition ϕ(x,y,z) = c, where c is a constant called the level-set value. Some of the common TPMS equations are described in Equations (1)–(4), which were found in the literature [16]:

(1) Primitive: cos(2απx)+cos(2βπy)+cos(2γπz)=c

(2) Gyroid: sin(2απx)cos(2βπy)+sin(2βπy)cos(2γπz)+sin(2γπz)cos(2απx)=c

(3) Diamond: cos(2απx)cos(2βπy)cos(2γπz)−sin(2απx)sin(2βπy)sin(2γπz)=c

(4) $$\mathrm{IWP}:\;\begin{array}{l} 2(\cos(2\alpha \pi x)\cos(2\beta \pi y))+\cos(2\beta \pi y)\cos(2\gamma \pi z)+\cos(2\gamma \pi z)\cos(2\alpha \pi x)-\\ (\cos2(2\alpha \pi x)+\cos2(2\beta \pi y)+\cos2(2\gamma \pi z))=c \end{array}$$

where α, β, and γ denote constants related to the unit cell size (L) in x, y, and z, respectively, and c is the offset parameter. A sheet structure has an offset parameter of nearly zero, while a network TPMS structure has ϕ(x,y,z) ≥ C or ϕ(x,y,z) ≤ C. Figure 2 shows two TPMS lattice (gyroid and primitive) and wavy-fin structures. Figure 2a–c shows computational domains, and Figure 2d–i shows examples of porous structures. As we can see from the figures, lattice structures have complex geometries and cannot be easily manufactured using every CAD tool. In this work, 3D geometries of TPMS were generated using MSLattice software version 1.0 [28]. The material of which the considered structures are composed is aluminum foam (Figure 2) with various porosities (from 0.1 to 0.9).

As illustrated in Table 1, among the HS structures with identical lattice thicknesses, the gyroid lattice exhibits the highest surface-to-volume ratio, while the wavy structure has the lowest. In the modeling of various lattice geometries, decreasing the unit length of a lattice causes its surface-to-volume ratio to increase linearly [29].

### 2.2. Geometric Modeling and Boundary Conditions

**Figure 3:**
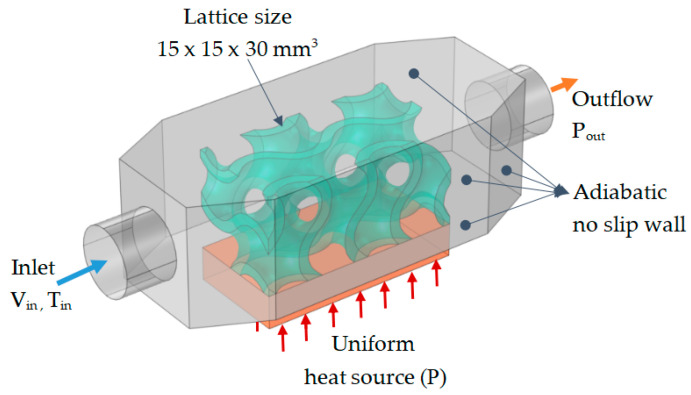
Simulation domains (grey—fluid channel, green—TPMS lattice structure, orange—heat source) and boundary conditions.

The full geometry of the model contains a fluid channel, a uniform source of heat from the bottom, and three different heat sink geometries (gyroid, primitive, and wavy—see Figure 2). The dimensions of all the heat sink structures are 15 × 15 × 30 [mm^3^], with a thickness of 2.5 [mm]. In order to allow the fluid to fully develop at both the inlet and outlet, a 15 [mm] extension section was added to the end structures. Additionally, cylindrical shapes were incorporated at both ends of the fluid channel model. To simplify the comparison of heat performances between the TPMS and the traditional wavy-fin heat sink, we assumed that the heat transfer area of the fin structure closely matches that of the gyroid lattice. The edges of channels were fileted to reduce pressure loss during analysis. The copper plate heat source, measuring 30 × 15 × 6 [mm^3^], is modeled at the bottom of the fluid domain. Figure 3 is a 3D model of the heat sink and the set of boundary conditions.

In this study, the heat transfer medium (water), with a constant inlet velocity, $V_{in}$, and inlet temperature, T_in_, flows into the heat sink structure, and the copper plate on the bottom generates heat uniformly at a constant power (P = 36 Watts). Induced heat is transferred to the shell and heat sink structures through conduction. Then, it dissipates into the environment via convection through the outlet region [30]. The outlet boundary is the pressure outlet, $P_{out}$. The outer walls of the channels were considered to be adiabatic with a no-slip condition in order to neglect the effects of heat transfer from the lateral boundaries. The other boundary conditions for heat transfer are insulation and fluid flow in non-slip conditions. The boundary conditions for the inlet and outlet are detailed in Table 2. Excellent-thermal-conductivity materials such as copper (heating plate) and aluminum (heat sink structure) were used to enhance overall heat dissipation. Table 3 details the fluid and thermal properties of heat sink materials [31]. In our study, the material properties were assumed to be constant. This assumption is justified because the temperatures considered in the analyses were relatively low (in the range of 20 to 100 °C). In this range, the temperature dependence of material properties can be considered negligible.

### 2.3. Governing Equations

A three-dimensional Multiphysics stationary problem built in COMSOL version 5.1 was selected to evaluate the heat transfer and fluid flow characteristics of gyroid, primitive TPMS, and wavy-fin heat sink structures. The simulation domain, created in MSLattice version 1.0 and saved in STL format, was imported into COMSOL and subsequently repaired. Computational fluid dynamics (CFD) simulations were utilized to analyze the structural and fluid flow behavior of the heat sinks. In the analysis, some necessary assumptions were made: (1) the fluid passing through the channel is Newtonian, incompressible, and steady-state; (2) no heat is generated inside the porous/foam medium; (3) there is a local thermal equilibrium between the fluid and solid phases of metal foam; and (4) it is reasonable to employ the laminar flow regime and Brinkman equation to compute the single-phase flow characteristics, as a laminar model provides reliable predictions of heat transfer rates and pressure drops in a heat sink that incorporates TPMS structures.

Considering the above assumptions, in COMSOL Multiphysics, the following built-in governing equations were used to compute heat transfer between the fluid and solid domains.

Fluid flow is governed by the Navier–Stokes equations:

(5) $$0=\nabla \cdot \left(-pI+\mu \left(\nabla u+{\left(\nabla u\right)}^{T}\right)+F\right),$$

(6) $$\rho \left(\nabla \cdot u\right)=0,$$

where **u** is the fluid velocity vector, ρ is the density of the fluid [kg/m^3^], and μ is dynamic viscosity [Pa·s].

The Brinkman equation was used to describe the flow of a fluid through a porous/foam medium in the channel [25,32]:

(7) $$\frac{\rho }{\varepsilon }\left(\left(u\cdot \nabla \right)\frac{u}{\varepsilon }\right)=\nabla \cdot \left(-pI+\frac{\mu }{\varepsilon }\left(\nabla u+{\left(\nabla u\right)}^{T}\right)-\left(\frac{\mu }{K}+\beta_{f}\left|u\right|\right)u+F\right),$$

(8) $$\nabla \cdot \left(\rho u\right)=0,$$

where ε is the porosity of the aluminum foam matrix, K is the permeability of aluminum foam, μ is the dynamic viscosity of water, $\beta_{f}$ represents the Forchheimer coefficient, and **F** is the buoyancy term.

The heat transfer in a solid was modeled using the energy equation:

(9) $$\nabla \cdot \left(-k_{s}\nabla T\right)=0.$$

The energy equation was also used to explain heat transfer inside the foam (Equation (10)) and fluid (Equation (11)), and it is expressed as follows:

(10) $$\rho c_{p}u\cdot \nabla T=\nabla \cdot \left(k_{eff}\nabla T\right),$$

(11) $$\rho c_{p}u\cdot \nabla T=\nabla \cdot \left(k_{f}\nabla T\right).$$

The effective thermal conductivity of a porous/foam medium filled with fluid can be described using the porosity (ε) and thermal conductivity of the solid phase ($k_{s}$) and fluid phase ($k_{f}$) via the following equation of the rule of mixtures [32,33]:

(12) $$k_{eff}=\varepsilon \cdot k_{f}+\left(1-\varepsilon \right)\cdot k_{s},$$

where **u** is the velocity of a fluid in the channel or porous/foam.

Accurate representation of the structural parameters of metal foam is important for estimating effective thermal conductivity. Calmidi and Mahajan [34] experimentally obtained the effective thermal conductivity of ERG aluminum foam using 1D heat conduction through a 2D foam structure. They reported an effective thermal conductivity value of 7.65 W/m·K at a porosity of 0.905. Boomsma and Poulikakos [35] extended this work with a 3D analytical model. They examined aluminum foam as tetradecahedron cells with cubic nodes at ligament intersections and reported that the effective thermal conductivity at a porosity of 0.9 was in the range of 7–9 W/m·K, depending on the value of the ratio of cubic node length at the intersection of two ligaments with respect to the ligament-to-ligament length ratio. Dai et al. [36] identified geometric and algebraic errors in Boomsma and Poulikakos’s [35] model, corrected them, and noted deviations from the experimental data. They further extended the model by including ligament inclination, and the results showed good agreement with the experiments (12.2% relative error). Yang et al. [37] designed an analytical and experimental model for determining the effective thermal conductivity of fluid-saturated metal foam with realistic node sizes. The analytical results agreed with the experimental results obtained by Calmidi and Mahajan [34]. The authors found that at a porosity of 0.9 and e = 0.3, the effective thermal conductivity was 8.5 W/m·K. They also reported that pore density had minimal effect on conductivity, and slight deviations were observed compared to the data obtained by Boomsma and Poulikakos [35] at e = 0.336. The effect of the effective thermal conductivity deviation of ERG aluminum foam on the average Nusselt number was numerically evaluated. The results showed that variations in effective thermal conductivity based on [35,37] had an insignificant effect on the average Nusselt number, with a relative error below 5%. In this work, Boomsma and Poulikakos’s [35] model was employed to evaluate the effective thermal conductivity.

In this study, the hydraulic parameters of the TPMS structures were analyzed using the Reynolds number, friction factor, and pressure drop. The Reynolds number was calculated to characterize the flow regime, and it is derived as follows [1,20]:

(13) Re=ρuDhμε,

where ρ is fluid density, D_h_ is the hydraulic diameter of the porous structure, u is the characteristic fluid velocity, μ is the viscosity of the fluid, and ε is porosity. The hydraulic diameter, $D_{h}$, for TPMS structures was determined as follows [38]:

(14) $$D_{h}=\frac{4v_{void}}{A_{s}},$$

where, $v_{void}$ represents void volume, and $A_{s}$ is the wetted surface area of the structure.

The heat transfer efficiency of the TPMS structure was evaluated using the convective heat transfer coefficient (h) and Nusselt number (Nu). The convective heat transfer coefficient indicates the rate at which heat is transferred by convection relative to conduction within a fluid, and it is calculated as follows [39]:

(15) $$h=\frac{q\prime \prime }{T-T_{\mathrm{in}}},$$

where q″ is heat flux applied at the bottom of the heat source. It is obtained by determining the heat generated (36 Watts) per cross-sectional area (15 [mm] × 30 [mm]), yielding 8 [W/cm^2^]. T is the local temperature measured on the copper plate 1 mm below the interface. The logmean temperature difference, $\Delta T_{\mathrm{LMTD}}$, can be determined as follows [38]:

(16) $$\Delta T_{\mathrm{LMTD}}=\frac{T_{\mathrm{out}}-T_{\mathrm{in}}}{\ln\left(\frac{T_{h}-T_{\mathrm{in}}}{T_{h}-T_{\mathrm{out}}}\right)}.$$

The local Nusselt number (Nu) characterizes the intensity of the transfer of heat from a fluid to a solid boundary and can be estimated using Equation [40]:

(17) $$\mathrm{Nu}=\frac{hD_{h}}{k},$$

where k is the thermal conductivity of water. The Darcy friction factor, f, and another dimensionless parameter are defined as follows [41]:

(18) $$f=\frac{\Delta pD_{h}}{2L\rho u^{2}}$$

where Δp, L, and u are pressure loss between the inlet and outlet; the length of the TPMS structure; and the inlet velocity, respectively. The overall performance evaluation criterion (PEC) was determined by combining the dimensionless parameter f and Nu. It is presented in the following equation:

(19) PEC=Nuavgf13.

### 2.4. Methods of Evaluating Mesh Independence

**Figure 4:**
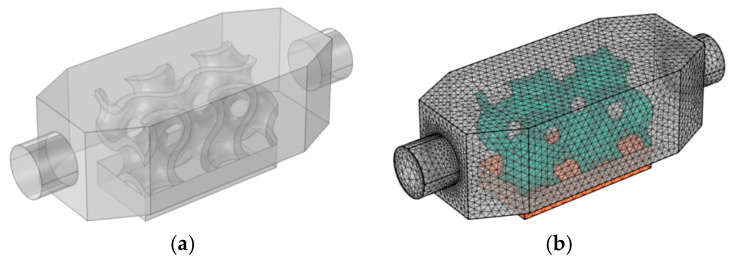
Three-dimensional (**a**) model and (**b**) meshes of heat sink domains (grey—fluid channel, green—TPMS lattice structure, orange—heat source).

A mesh independence check was performed using COMSOL Multiphysics, incorporating specific meshing strategies such as corner refinement, free tetrahedral elements, and boundary layers. An unstructured free tetrahedral was used for discrete complex TPMS structures, while a hexahedral mesh was used for the rectangular box of the heat source. Three computational meshing domains (hot plate, heat sink, and fluid domain) are explained in Figure 4. The mesh is denser within the internal TPMS structure relative to the outer surface, and it helps capture complex flow and heat transfer phenomena. Mesh convergence testing (via a grid independence study) was conducted to validate the accuracy of the computed flow and thermal fields. Table 4 details average Nusselt number (${Nu}_{avg}$) computational results at different mesh sizes. The optimum Nu_avg_ was obtained at a finer mesh size with 504,487 domain elements; it was used in temperature, velocity, and pressure calculations.

## 3. Results and Discussion

### 3.1. Fluid Flow Characteristics

**Figure 5:**
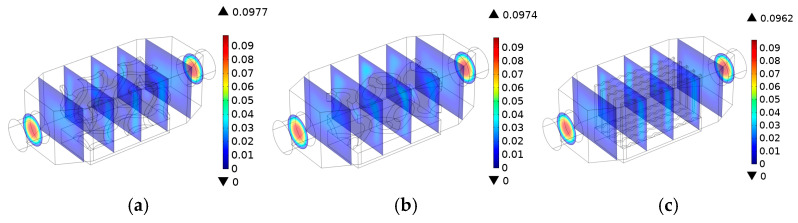
Flow field distribution profiles: (**a**) gyroid, (**b**) primitive, and (**c**) wavy.

Figure 5 presents the velocity map of the heat transfer medium within lattice (gyroid and primitive) and traditional wavy-fin models. The figure shows that the complex contour surfaces of the model greatly influence flow characteristics. The lengths of the cross-sections were determined transversely, with dimensions in millimeters, and found to be 5, 15, 25, 35, 45, 55, and 65 in the direction of fluid flow. The results indicated that, in comparison to wavy and primitive structures, the gyroid TPMS displays a distinct flow pattern, with a slightly higher flow velocity (0.0977 [m/s]) distributed non-uniformly within the channel. In the gyroid lattice, turbulent flow characteristics and a high surface area were observed, leading to high heat transfer. In the primitive TPMS, the high-velocity area is concentrated near the center of the channel (37.5 [mm]), while a wavy flow is distributed uniformly along the parallel path. Less effective heat transfer relative to the TPMS heat sink was observed in the traditional heat sink (wavy fin).

**Figure 6:**
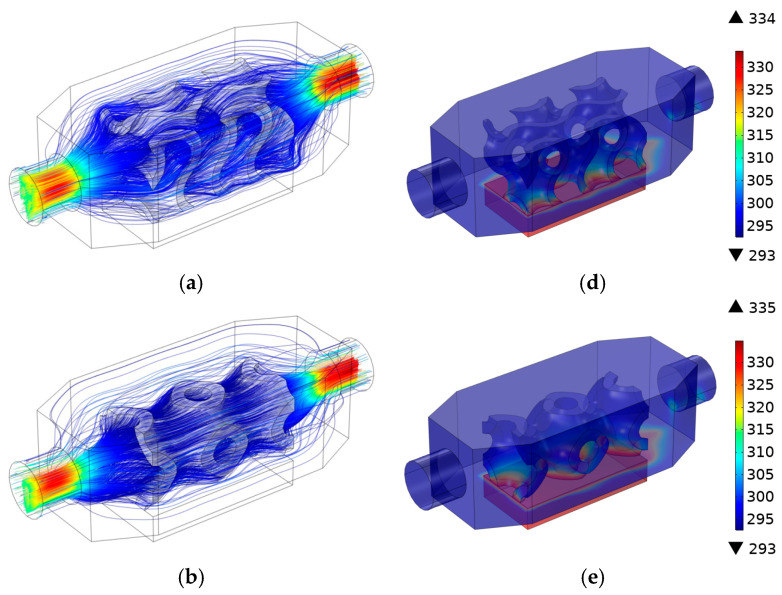

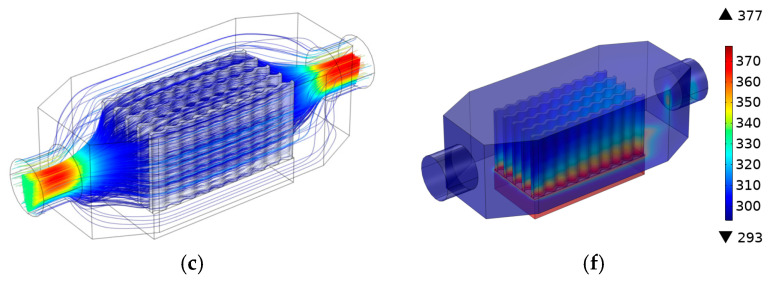
Streamline velocity profiles and temperature distributions for different HS geometries: (**a**) velocity profile—gyroid, (**b**) velocity profile—primitive, (**c**) velocity profile—wavy, (**d**) temperature profile—gyroid, (**e**) temperature profile—primitive, and (**f**) temperature profile—wavy.

Figure 6 presents streamline velocity and temperature distribution profiles for the different heat sink models. In the case of the velocity profiles, the most striking feature pertaining to the gyroid structure is the highly intricate and tortuous flow paths. Within this lattice, the streamlines are characterized by twists, turns, and interconnected passages distributed throughout the entire volume of the heat sink. Despite the complexity of the paths, the uniform streamlines filled the channels, helping to generate effective fluid distribution and maximize the heat transfer area (Figure 6a).

The primitive lattice exhibits streamlines that are denser around its primitive cells. This configuration results in less tortuous flow paths and a consequently lower pressure drop relative to the gyroid structure (Figure 6b). The smooth and less convoluted parallel path of the wavy fin lowers the pressure drop relative to the gyroid and primitive lattices (Figure 6c). Variation in temperature distribution is visible in different heat sink geometries, as shown in Figure 6d–f. At the opening of the inner structure, the heat source from bottom plate triggered a rapid increase in temperature. Eventually, the temperature reached its peak and began to slightly decline when moving past the end of the structure. Among these models, the max temperature of 377 [K] was obtained from the wavy-type heat sink. The primitive lattice exhibits intermediate temperatures between those of the wavy and gyroid models and performs better in terms of heat dissipation than the wavy structure. The gyroid structure consistently maintains the lowest temperature, thereby facilitating more efficient transfer of heat away from the heat sources.

### 3.2. Effects of Porosity on Temperature Distribution Across the Length of the Channel

**Figure 7:**
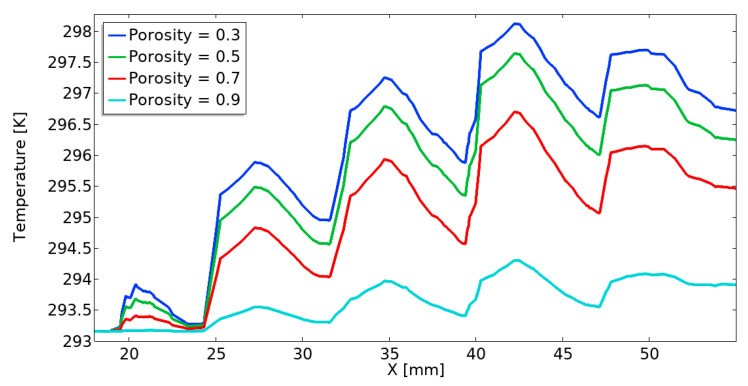
Effects of porosity on temperature profiles along the length of the channel.

Figure 7 presents the temperature profile along the line passing through the center of the heat sink channel for porosity levels varying from 0.3 to 0.9, with constant permeability at 1 × 10^−8^ [m^2^]. The temperature distribution exhibits a wave-like pattern due to the intricate geometry of the gyroid heat sink structure. Evidently, the temperature result decreases with an increase in the porosity level. Notably, the highest temperature occurs at a porosity of 0.3, while the lowest temperature can be observed at a porosity of 0.9. This result indicates that a higher porosity value results in a higher fluid volume fraction and a reduced solid fraction, providing a larger flow passage, thereby allowing more coolant to move freely through the porous structure and improving heat extraction from the heated surface. However, a higher porosity value reduces the effective thermal conductivity of the solid matrix; in the plot below, the convective cooling effect dominates, resulting in a decrease in temperature.

### 3.3. Log-Mean Temperature Difference (LMTD) in the Heat Sink

**Figure 8:**
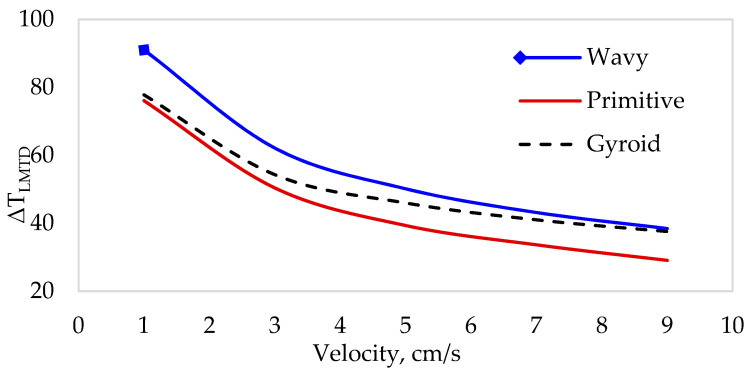
Log-mean temperature difference vs. flow velocities.

Figure 8 illustrates fluid flow velocity vs. the log-mean temperature difference, $\Delta T_{LMTD}$, between the inlet and outlet boundaries of the heat sink. As fluid velocity increases, the convective heat transfer coefficient rises, and the surface of the heat sink cools more effectively. This reduces the temperature gradient between the heat sink and the fluid, resulting in faster dissipation of heat from the heat sink. This finding reveals that the primitive heat sink design exhibits a lower LMTD than both the gyroid and wavy structures.

**Figure 9:**
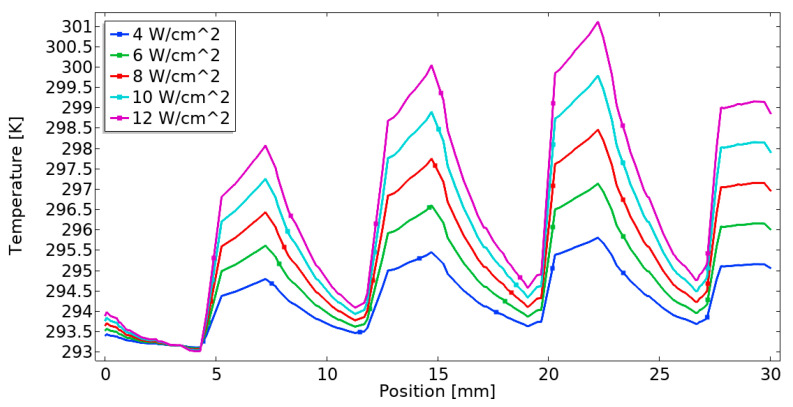
Heat flux effect on the temperature profile along channel length.

Figure 9 presents the heated temperature distribution along the line passing through the center of the channel under different heat fluxes (4 [W/cm^2^], 6 [W/cm^2^], 8 [W/cm^2^], 10 [W/cm^2^], and 12 [W/cm^2^]), with an inlet water fluid velocity of 0.05 [m/s] at a constant porosity of 0.9 and a permeability of 1 × 10^−8^ [m^2^]. The temperature in the gyroid lattice structure is higher in the direction of fluid flow, indicating that the heat generated by the copper block was transferred into the working fluid quickly due to the high heat transfer performance of the foam TPMS heat sink. The simulation results show that the maximum temperature of 302 [K] at a heat flux of 12 [W/cm^2^] occurs around the end edge of the heat source (23.5 [mm]). This result indicates that an increase in the heat source from the bottom plate leads to an increase in the temperature distribution.

### 3.4. Pressure Gradient in Different HS Geometries

**Figure 10:**
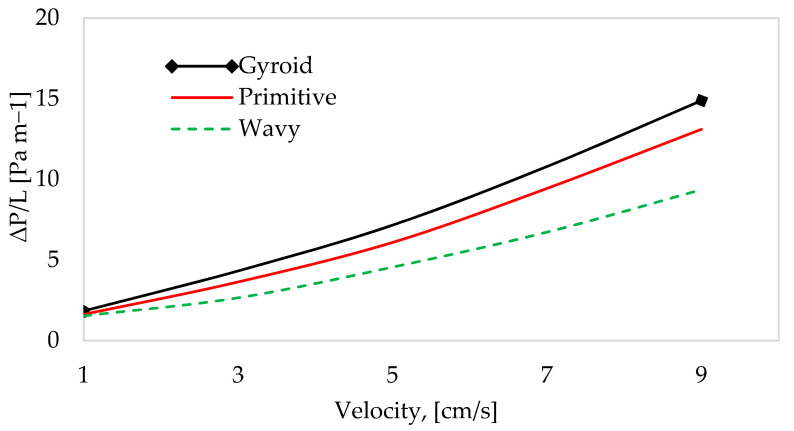
Pressure gradient vs. fluid flow velocity.

Figure 10 presents a comparison of the pressure drop per unit length for the gyroid, primitive, and wavy structures. In all the models, increasing flow velocity raises the pressure gradient due to the greater frictional losses induced inside the structure. In the context of geometry, giving the gyroid structure an intricate and convoluted shape leads to a greater pressure drop relative to the other heat sink structures. In terms of geometry, the intricate and convoluted shape of the gyroid structure leads to a higher pressure drop. However, in the traditional wavy-fin design, the flow of fluid through the parallel channels between the fins results in reduced flow resistance. This observation was made by Samson et al. [42], and it is proven again in this study.

### 3.5. Local Nusselt Number Distribution Along the Length of the Channel

**Figure 11:**
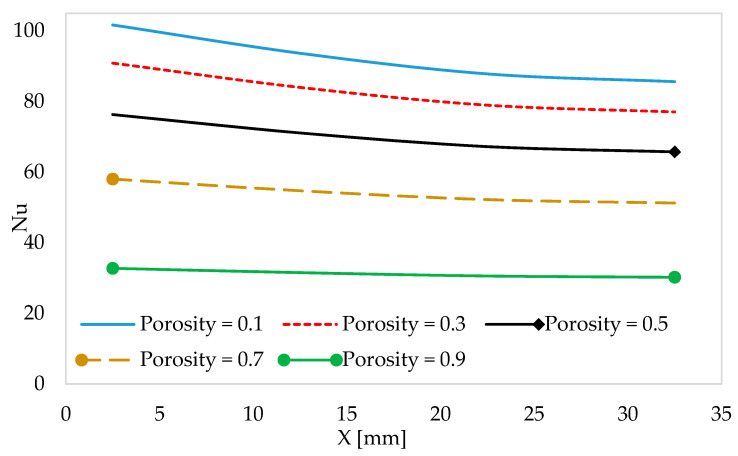
The influence of porosity on the Nusselt number along the channel section.

Figure 11 compares the Nusselt number for the gyroid, primitive, and wavy-fin models at different porosities ranging from 0.1 to 0.9. In all cases, the Nusselt number slightly declines with the increase in the length of the channel. It is evident from this result that, at lower porosities, a greater surface area promotes higher Nusselt numbers and ensures flow resistance is high, thereby limiting fluid penetration, while at higher porosities, a reduced solid fraction lowers heat transfer. This is because the surface area decreases with an increase in porosity. For the TPMS heat sink, the best porosity range is typically 0.5 to 0.7, wherein cooling performance and pumping power (hydraulic performance) are balanced.

**Figure 12:**
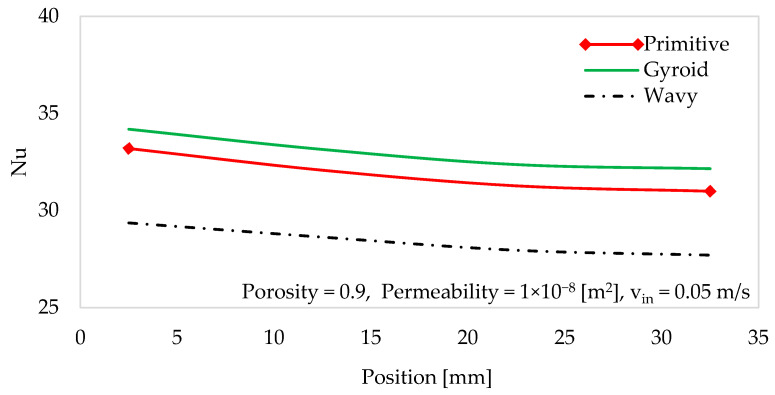
Profile of the Nusselt number along the channel.

Nusselt number variations are strongly influenced by heat sink geometry. In Figure 12, the effects of three different heat sink geometries on the Nusselt number are compared under conditions of a porosity of 0.9 and a permeability of 1 × 10^−8^ [m^2^]. In all the models, the Nusselt number decreases with an increase in channel length, because of the thermal resistance that develops, which reduces the local heat transfer coefficient. These results clearly indicate that the gyroid structure exhibits the highest Nusselt number among the geometries tested (primitive and wavy-fin geometries). The complex and interconnected network gyroid structure creates a highly tortuous flow path, promoting greater fluid mixing and proving the most effective for heat removal.

### 3.6. Comparison of the Thermal Performances of the Wavy-Fin and TPMS (Gyroid and Primitive) Heat Sinks

**Figure 13:**
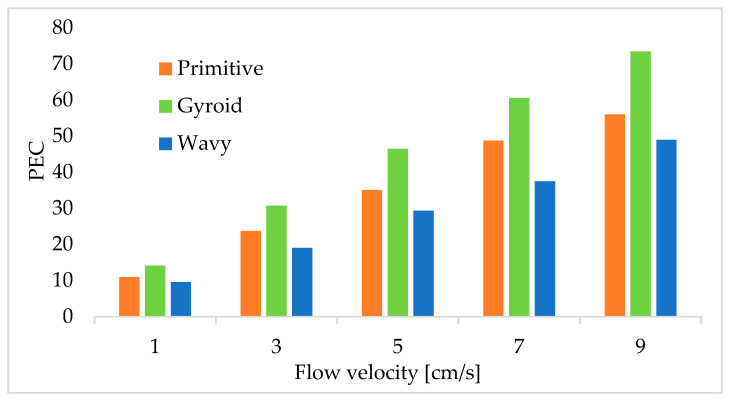
Variation in the performance evaluation criterion with different heat sink designs.

The performance evaluation criterion (PEC) of TPMS lattice and traditional wavy-fin heat sinks was determined using Equation (19), and the results are illustrated in Figure 13. As shown in the bar chart, as the fluid flow velocity increased, the PEC values for all the models also increased. The increased flow velocity makes it easier to carry heat away from the source. As indicated in the figure, the gyroid TPMS porous heat sink achieved the highest thermal performance evaluation criterion among the heat sink models tested. Moreover, an interesting finding revealed that the highest PEC, 73.39, was obtained from the porous gyroid heat sink at a flow velocity of 5 cm/s. This result is in good agreement with previous numerical and experimental studies. Yeranee (2022) [16] and Saghir (2024) [43] reported that gyroid TPMS structures exhibit a high performance evaluation criterion. This finding confirms that a porous gyroid TPMS is the most effective structure for heat sink applications.

## 4. Conclusions

Triply periodic minimal surface (TPMS) structures have drawn a great deal of attention from researchers in regard to heat sink applications. The advantage of these structures lies in their compact design and significant thermo-physical properties. In this work, the heat transfer performance and flow characteristics of two porous TPMS lattices (gyroid and primitive) were compared with those of a traditional wavy-fin heat sink using the finite element numerical method. This structure has a high surface area-to-volume ratio, which is the basic requirement for thermal management. Water was used as the fluid medium in the analysis, and aluminum served as the material for the foam TPMS structure. Based on the simulation results, the flow behavior, heat transfer characteristics, and thermal performances of porous TPMS lattices were investigated. The following conclusions were drawn from our findings.

(1) Compared to the traditional wavy-fin structure, the porous TPMS lattice exhibits a higher Nusselt number under the same inlet conditions, including with respect to porosity, permeability, inlet velocity, and inlet temperature. Additionally, the average Nusselt number decreased with the increase in the length of the channel in all heat sink models due to the increasing local temperature.
(2) Among the three structures, the porous gyroid TPMS exhibited the highest pressure gradient. This behavior is primarily attributed to its intricate and highly convoluted geometry, which increases frictional losses within a porous medium.
(3) The temperature profile of the heat sink structure deteriorated with an increase in porosity because a high porosity value allows for larger flow passages that allow greater flow of coolant through the porous structure, enhancing the extraction of heat from the heated surface. However, an increase in porosity leads to lower effective thermal conductivity, which is an important parameter for determining overall thermal performance.
(4) The porous gyroid TPMS structure achieved the highest performance evaluation criterion (PEC) value of 73.39 at a flow velocity of 5 cm/s. Moreover, the gyroid porous structure with a porosity of 0.5 to 0.7 exhibited balanced hydraulic performance and better cooling performance in heat sink analysis. This research summarizes the potential applications of porous TPMS lattice structures in thermal management.

In future work, experimental investigations can be conducted to validate these results and further evaluate the overall heat transfer performance of these models.

## Figures and Tables

**Table 1:** Geometric characteristics of various heat sink structures.

Type	SA	SA to V Ratio	Lattice Length, [mm]
Primitive	4288	2.32	15 × 15 × 30
Gyroid	5183	2.82	
Wavy fin	9496	1.86	

**Table 2:** Boundary conditions for the inlet and outlet.

Boundary	Temperature	Velocity	Pressure
Inlet	T_in_ = 293.15 [K]	$u=V_{in}$ v=w=0	-
Outlet	-	-	$P_{\mathrm{out}}=0$.

**Table 3:** Fluid and thermal properties of the materials.

Materials	Thermal Conductivity [W/(m K)]	Density [kg/m^3^]	Specific Heat Capacity [J/(kg K)]	Dynamic Viscosity [kg/(m s)]
Copper	400	8960	385	-
Aluminum	238	2700	900	-
Water	0.6	997	4182	0.89 × 10^−3^

**Table 4:** Mesh sensitivity analysis for the average Nusselt number.

Mesh Size	Domain Elements	Boundary Elements	Edge Elements	Average Nusselt Number
Extra coarser	20,866	4813	693	52.3
Coarser	27,225	5901	786	54.9
Normal	114,512	14,175	1228	60.9
Fine	195,069	21,295	1532	61.7
Finer	504,487	39,930	2101	63.4

## Data Availability

The original contributions presented in this study are included in the article. Further inquiries can be directed to the corresponding authors.

## References

[1] Tang W., Zhou H., Zeng Y., Yan M., Jiang C., Yang P., Li Q., Li Z., Fu J., Huang Y. (2023). Analysis on the convective heat transfer process and performance evaluation of Triply Periodic Minimal Surface (TPMS) based on Diamond, Gyroid and Iwp. Int. J. Heat Mass Transf..

[2] Gaikwad A., Sathe A., Sanap S. (2023). A design approach for thermal enhancement in heat sinks using different types of fins: A review. Front. Therm. Eng..

[3] Behnia M., Copeland D., Soodphakdee D. A comparison of heat sink geometries for laminar forced convection: Numerical simulation of periodically developed flow. Proceedings of the Sixth Intersociety Conference on Thermal and Thermomechanical Phenomena in Electronic Systems.

[4] Wang Q., Tao J., Cui Z., Zhang T., Chen G. (2024). Numerical simulation of fluid and heat transfer characteristics of microchannel heat sink with fan-shaped grooves and triangular truncated ribs. Int. Commun. Heat Mass Transf..

[5] Zohora F.T., Haque M.R., Haque M.M. (2023). Numerical investigation of the hydrothermal performance of novel pin-fin heat sinks with hyperbolic, wavy, and crinkle geometries and various perforations. Int. J. Therm. Sci..

[6] Soodphakdee D., Behnia M., Copeland D.W. (2001). A comparison of fin geometries for heatsinks in laminar forced convection: Part I-round, elliptical, and plate fins in staggered and in-line configurations. Int. J. Microcircuits Electron. Packag..

[7] Jajja S.A., Ali W., Ali H.M., Ali A.M. (2014). Water cooled minichannel heat sinks for microprocessor cooling: Effect of fin spacing. Appl. Therm. Eng..

[8] Yaseen S.J. (2023). Numerical study of the fluid flow and heat transfer in a finned heat sink using Ansys Icepak. Open Eng..

[9] Mrozek A., Strek T. (2022). Numerical Analysis of Dynamic Properties of an Auxetic Structure with Rotating Squares with Holes. Materials.

[10] Mrozek-Czajkowska A., Stręk T. (2024). Design Optimization of the Mechanics of a Metamaterial-Based Prosthetic Foot. Materials.

[11] Han D., Ren X., Zhang Y., Zhang X.Y., Zhang X.G., Luo C., Xie Y.M. (2022). Lightweight auxetic metamaterials: Design and characteristic study. Compos. Struct..

[12] Novak N., Vesenjak M., Kennedy G., Thadhani N., Ren Z. (2019). Response of Chiral Auxetic Composite Sandwich Panel to Fragment Simulating Projectile Impact. Phys. Status Solidi.

[13] Burlaga B., Kroma A., Poszwa P., Kłosowiak P., Popielarski P., Stręk T. (2022). Heat Transfer Analysis of 3D Printed Wax Injection Mold Used in Investment Casting. Materials.

[14] Grima-Cornish J.N., Attard D., Grima J.N., Evans K.E. (2022). Auxetic Behavior and Other Negative Thermomechanical Properties from Rotating Rigid Units. Phys. Status Solidi RRL.

[15] Attarzadeh R., Rovira M., Duwig C. (2021). Design analysis of the “Schwartz D” based heat exchanger: A numerical study. Int. J. Heat Mass Transf..

[16] Yeranee K., Rao Y. (2022). A review of recent investigations on flow and heat transfer enhancement in cooling channels embedded with triply periodic minimal surfaces (TPMS). Energies.

[17] Singh A., Al-Ketan O., Karathanasopoulos N. (2023). Mechanical performance of solid and sheet network-based stochastic interpenetrating phase composite materials. Compos. Part B Eng..

[18] Anacreonte A.V., Iasiello M., Mauro G.M., Bianco N., Chiu W.K. (2025). Pore-scale multi-objective shape optimization of triply periodic minimal surface cellular architectures: Volumetric Nusselt number versus friction factor. Int. J. Heat Mass Transf..

[19] Liu J., Cheng D., Oo K., McCrimmon T.L., Bai S. (2024). Design and Additive Manufacturing of TPMS Heat Exchangers. Appl. Sci..

[20] Liu Z., Gao Z., Dai M., Song B., Yang B., Zhang T., Yuan S., Liu G., Zhao M. (2025). Fluid Flow and Heat Transfer Performances of Aluminum Alloy Lattices with Triply Periodic Minimal Surfaces. Materials.

[21] Baobaid N., Ali M.I., Khan K.A., Al-Rub R.K.A. (2022). Fluid flow and heat transfer of porous TPMS architected heat sinks in free convection environment. Case Stud. Therm. Eng..

[22] Oh S.H., Kim J.E., Jang C.H., Kim J., Park C.Y., Park K. (2025). Multifunctional gradations of TPMS architected heat exchanger for enhancements in flow and heat exchange performances. Sci. Rep..

[23] Beer M., Rybár R. (2024). Numerical Study of Fluid Flow in a Gyroid-Shaped Heat Transfer Element. Energies.

[24] Barakat A., Sun B. (2024). Controlling TPMS lattice deformation for enhanced convective heat transfer: A comparative study of Diamond and Gyroid structures. Int. Commun. Heat Mass Transf..

[25] Beer M., Rybár R. (2024). Optimisation of Heat Exchanger Performance Using Modified Gyroid-Based TPMS Structures. Processes.

[26] Tang W., Guo J., Yang F., Zeng L., Wang X., Liu W., Zhang J., Zou C., Sun L., Zeng Y. (2024). Performance analysis and optimization of the Gyroid-type triply periodic minimal surface heat sink incorporated with fin structures. Appl. Therm. Eng..

[27] Al-Ketan O., Ali M., Khalil M., Rowshan R., Khan K.A., Abu Al-Rub R.K. (2021). Forced convection computational fluid dynamics analysis of architected and three-dimensional printable heat sinks based on triply periodic minimal surfaces. J. Therm. Sci. Eng. Appl..

[28] Al-Ketan O., Abu Al-Rub R.K. (2021). MSLattice: A free software for generating uniform and graded lattices based on triply periodic minimal surfaces. Mat. Des. Process Comm..

[29] Dutkowski K., Kruzel M., Rokosz K. (2022). Review of the state-of-the-art uses of minimal surfaces in heat transfer. Energies.

[30] Lin L., Zhao J., Lu G., Wang X.D., Yan W.M. (2017). Heat transfer enhancement in microchannel heat sink by wavy channel with changing wavelength/amplitude. Int. J. Therm. Sci..

[31] Small E., Sadeghipour S.M., Asheghi M. (2006). Heat sinks with enhanced heat transfer capability for electronic cooling applications. J. Electron. Packag..

[32] Bayomy A.M., Saghir Z. (2020). Thermal performance of finned aluminum heat sink filled with ERG aluminum foam: Experimental and numerical approach. Int. J. Energy Res..

[33] Bayomy A., Saghir M. (2017). Experimental and Numerical Study of the Heat Transfer Characteristics of Aluminium Metal Foam (with/without channels) Subjected to Steady Water Flow. Pertanika J. Sci. Technol..

[34] Calmidi V.V., Mahajan R. (1999). The effective thermal conductivity of high-porosity fibrous metal foams. J. Heat Transf..

[35] Boomsma A., Poulikakos D. (2001). On the effective thermal conductivity of a three-dimensionally structured fluid-saturated metal foam. Int. J. Heat Mass Transf..

[36] Dai Z., Nawaz K., Park Y., Bock J., Jacobi A. (2010). Correcting and extending the Boomsma-Poulikakos effective thermal conductivity model for three-dimensional, fluid-saturated metal foams. Int. Commun. Heat Mass Transf..

[37] Yang X.H., Bai J.X., Yan H.B., Kuang J.J., Lu T.J., Kim T. (2014). An analytical unit cell model for the effective thermal conductivity of high porosity open-cell metal foams. Trans. Porous Media.

[38] Barakat A., Pan Y., Sun B. Comparative Heat Transfer Performance of TPMS Structures: Spotlight on Fisch Koch S versus Gyroid, Diamond and SplitP Lattices. Proceedings of the 10th International Conference on Energy Materials and Environment Engineering (ICEMEE 2024).

[39] Arshad A., Saeed M., Ikhlaq M., Imran M., Yan Y. (2025). Heat and fluid flow analysis of micro-porous heat sink for electronics cooling: Effect of porosities and pore densities. Therm. Sci. Eng. Prog..

[40] Saghir M.Z., Rahman M.M. (2024). Effectiveness in Cooling a Heat Sink in the Presence of a TPMS Porous Structure Comparing Two Different Flow Directions. Fluids.

[41] Zhang Y., Peng F., Jia H., Zhao Z., Wang P., Duan S., Lei H. (2025). Conformal geometric design and additive manufacturing for special-shaped TPMS heat exchangers. Int. J. Heat Mass Transf..

[42] Samson S., Tran P., Marzocca P. (2023). Design and modelling of porous gyroid heatsinks: Influences of cell size, porosity and material variation. Appl. Therm. Eng..

[43] Saghir M.Z., Yahya M. (2024). Convection heat transfer and performance analysis of a triply periodic minimal surface (TPMS) for a novel heat exchanger. Energies.

